# Insight into Reepithelialization: How Do Mesenchymal Stem Cells Perform?

**DOI:** 10.1155/2016/6120173

**Published:** 2015-12-06

**Authors:** Deyun Chen, Haojie Hao, Xiaobing Fu, Weidong Han

**Affiliations:** Institute of Basic Medicine Science, Chinese PLA General Hospital, Beijing 100853, China

## Abstract

Wound reepithelialization is a cooperative multifactorial process dominated by keratinocyte migration, proliferation, and differentiation that restores the intact epidermal barrier to prevent infection and excessive moisture loss. However, in wounds that exhibit impaired wound healing, such as chronic nonhealing wounds or hypertrophic scars, the reepithelialization process has failed. Thus, it is necessary to explore a suitable way to mitigate these abnormalities to promote reepithelialization and achieve wound healing. Mesenchymal stem cells (MSCs) have the capacity for self-renewal as well as potential multipotency. These cells play important roles in many biological processes, including anti-inflammation, cell migration, proliferation, and differentiation, and signal pathway activation or inhibition. The mechanism of the involvement of MSCs in reepithelialization is still not fully understood. An abundance of evidence has shown that MSCs participate in reepithelialization by inhibiting excessive inflammatory responses, secreting important factors, differentiating into multiple skin cell types, and recruiting other host cells. This review describes the evidence for the roles that MSCs appear to play in the reepithelialization process.

## 1. Introduction

Wound healing is a highly coordinated and orderly process that requires the activities of different cell types, including inflammatory cells, keratinocytes, fibroblasts, and endothelial cells (ECs) [1]. It is mainly divided into three successive but partially overlapping phases: inflammation, reepithelialization/granulation tissue generation, and tissue remodeling [2]. During these wound healing phases, the restoration of epidermal integrity, also called reepithelialization, is essential to complete wound repair. A wound cannot be considered closed if reepithelialization is lacking regardless of the complete restoration of the underlying tissue [3].

In the reepithelialization phase of wound healing, epithelial cells migrate to the wound site, cover the granulation tissue, and then meet in the middle, at which point contact inhibition causes them to stop migrating, completing the reepithelialization [4]. However, perfect reepithelialization during wound healing remains a challenge for diabetic patients, surgical patients, and burn victims [5]. The pathologies of wound healing can be grouped into two categories: excessive wound healing, leading to hypertrophic scars, and incomplete wound healing, leading to chronic wounds [6]. These abnormalities most commonly occur in conjunction with impaired reepithelialization. Therefore, it is necessary to develop methods to attenuate these abnormalities to promote reepithelialization and achieve complete wound healing.

The current data indicate that the involvement of MSCs in the reepithelialization process is a promising solution for wounds with impaired healing due to diabetes, trauma, burns, and numerous other conditions. At the wound site, MSCs contribute to the generation of well-vascularized granulation tissue, promote reepithelialization, and attenuate scar formation by several mechanisms, including modulation of the inflammatory environment, enhancement of angiogenesis, promotion of the migration of keratinocytes, and recruitment of other host cells [7, 8]. Thus, the therapeutic application of MSCs has been shown to enhance wound reepithelialization and accelerate wound healing.

In this review, we summarize the current information regarding the role of MSCs, specifically in the reepithelialization process of wound repair, and their potential clinical applications.

## 2. Reepithelialization

Reepithelialization is a key component of wound closure. A skin wound cannot be considered closed if the reepithelialization has not occurred. The reepithelialization process involves the formation of new epithelium and skin appendages by activating the proliferation, migration, and differentiation of keratinocytes and reconstituting the protection of the underlying dermal structures [9]. In the reepithelialization process, keratinocytes reepithelialize the wound through their enhanced migration and mitosis at the wound periphery in the epidermis. Furthermore, fibroblasts migrate beneath the wound site to close the wound [10]. Once the wound area is covered, contact inhibition causes them to stop migrating and triggers the differentiation of the keratinocytes into stratified squamous keratinizing epidermal cells [11]. Keratinocytes, the predominant cellular component of the epidermis, are derived from epithelial stem cells (EpSCs), which are mainly located in the bulge of the hair follicle (HF) and the basal layer of the interfollicular epidermis (IFE) [12, 13]. After epidermal injury, EpSCs from both the HF and IFE niches give rise to the keratinocytes that migrate and reepithelialize the wound [14, 15]. Studies have reported that IFE EpSCs are major contributors toward the long-term repair of the epidermis [16]. HF bulge stem cells also migrate into the IFE to regenerate the epidermis immediately after injury, but this effect is temporary, suggesting that HF EpSCs provide an initial burst in the wound healing rate of defective shallow epidermis but are probably less important in larger wounds as well as in wounds that are difficult to heal and where reepithelialization cannot occur spontaneously [17, 18]. Therefore, abnormalities in the wound healing process result in various pathologies ranging from chronic wounds to hypertrophic scars, both of which display an impaired reepithelialization [13]. A better understanding of the impaired epithelialization process may provide insights into new therapeutic approaches to enhance reepithelialization and accelerate wound closure.

## 3. Impaired Reepithelialization: Chronic Wounds and Hypertrophic Scars

The ideal end-point of skin wound healing is the normal scar formation. However, abnormalities in this process lead to a series of pathologies from chronic wounds to hypertrophic scars, and these pathologies most commonly occur when reepithelialization has been delayed. A chronic wound that has failed to progress through the normal healing process may enter a persistent inflammatory state and a perpetual nonhealing state that is characterized by chronicity and frequent relapse. This is the first condition that was observed to exhibit an impaired reepithelialization process [19] and can be caused by various pathological conditions, including diabetes, trauma, burns, and numerous other conditions [20]. In chronic wounds, the delay of reepithelialization may be due to bacterial infection, tissue hypoxia, local ischemia, exudates, and excessive levels of inflammatory cytokines that create a continuous state of inflammation. Also in this state, the cell pool is impaired and may demonstrate increased cellular senescence and a decreased cellular response to growth factors [21, 22].

Furthermore, in chronic wounds, inflammatory cells, such as neutrophils, macrophages, and lymphocytes, which are sequentially recruited into the wound, are disrupted in this sequential process in the persistent inflammatory environment [1]. Delayed neutrophil and monocyte infiltration but more sustained lymphocyte infiltration has been considered to be an important factor of chronic wounds [20]. In addition, neutrophils release various enzymes, including collagenase, which degrades the extracellular matrix (ECM), and elastase, which destroys prohealing factors. This inflammatory environment also leads to impaired cellular functions of keratinocytes, ECs, and fibroblasts due to the excessive levels of the inflammatory cytokines; degradation of the ECM due to high matrix metalloproteinase (MMP) activity; inhibition of prohealing factors, which further recruit neutrophils and continue the above cycle [19]. An uncontrolled and continuous inflammatory response prevents wound healing. This observation contributes to an explanation of the delay in reepithelialization [23].

However, in some pathological situations, excessive wound healing may result in hypertrophic scars with serious cosmetic and functional implications, as well as decreased tensile strength compared to the surrounding normal skin [24]. This condition is also the result of impaired reepithelialization. Studies have shown that when wounds epithelialize in less than 10 to 14 days, there is almost no hypertrophic scar formation. However, when the wound epithelialization requires between 2 an 3 weeks, one-third of the wounds form hypertrophic scars, and wound epithelialization that takes more than 3 weeks results in a 78% rate of hypertrophic scarring [25]. Hypertrophic scar formation is directly caused by an unusual proliferation of fibroblasts and the deposition of excess ECM by the fibroblasts and myofibroblasts at the wound site [26]. Studies suggest that the keratinocytes in the epidermis of hypertrophic scars become activated and produce growth factors that affect the inflammatory response, endothelial cells, and fibroblasts. Abnormal proliferation and differentiation of the keratinocytes cause an increase in epidermal thickness and lead to hypertrophic scars formation [27]. In addition, an abnormally low rate of cell death and a high rate of fibroblast proliferation can also promote scar formation. In addition, fibroblasts become highly proliferative when cultured with keratinocytes, which demonstrates the positive role of keratinocytes in promoting fibroblast proliferation. Furthermore, myofibroblasts, which contribute to the composition, organization, and mechanical properties of ECM, increase collagen synthesis and inhibit cell migration, processes that also lead to scar formation [25, 27, 28].

The current treatments for chronic wounds are antibiotic treatment, pressure therapy, hyperbaric oxygen therapy, and revascularization therapy [29]. For hypertrophic scars, the treatments include surgical excision, pressure therapy, laser therapy, and therapies directed against collagen synthesis [30]. However, to date, optimal means to treat these conditions have not been identified. Therefore, it is necessary to explore new therapeutic approaches to solve the problems of chronic wounds and hypertrophic scars to enhance reepithelialization and accelerate wound closure.

## 4. Mesenchymal Stem Cells in Reepithelialization

Mesenchymal stem cells (MSCs) residing in the bone marrow were first described by Friedenstein et al. [31]. These cells comprise a small fraction (<0.1%) of the adult bone marrow cells [32]. Because of the lack of a specific single mesenchymal cell marker, MSCs are identified through a combination of physical, phenotypic, and functional characteristics. The International Society for Cellular Therapy proposed three minimal criteria to define MSCs: plastic adherent ability, expression of CD105, CD73, and CD90, lack of the expression of CD45, CD34, CD11b, CD79*α* or CD19, and HLA-DR, and a multilineage differentiation potential [33].

Bone marrow mesenchymal stem cells (BMSCs) are adult stem cells derived from mesodermal cell lineages [34]. Recent studies have proposed the three possible histological origins of MSCs. The first of these is the epithelial to mesenchymal transition (EMT), which is a cellular process in which epithelial cells lose their epithelial cell properties and acquire mesenchymal cell properties. Second, MSCs may have a potential perivascular origin, such as pericytes and avascular tissue, both of which are sources of MSCs. The third possibility is that they are derived from the human vascular adventitial fibroblasts in pulmonary arteries [35].

The commonly reported methods for isolation of BMSCs included untreated whole bone marrow (BM) blood adherent culture methods and density-gradient centrifugation methods. The untreated whole BM blood adherent culture methods are based on the BMSCs plastic adherence properties, whereas the density-gradient centrifugation methods are based on the suspension density of the BM cells. Density-gradient centrifugation methods may affect the BMSCs proliferation, but the BM blood adherent culture methods have no effect on heterogeneity and differentiation of the BMSCs. The BM blood adherent culture method may be an efficient method for isolation and purification of BMSCs [36].

After MSCs were originally isolated from bone marrow, MSCs were identified in various tissues, including adipose tissue [37], umbilical cord [38], muscles [39], amniotic fluid [40], and others [41]. These cells have raised great expectations in the field of regenerative medicine due to their straightforward isolation and expansion, unique anti-inflammatory and immunomodulatory properties, and potential multipotency [42]. In recent years with the rapid development of stem cell biology, MSCs have proven to be an attractive cell type for use as wound repair therapeutics. Studies have shown that MSCs may migrate to the wound site where they direct inflammation and antimicrobial activity, promote cell migration, proliferation and differentiation, and recruit other host cells to play critical roles in reepithelialization and wound repair [43].

### 4.1. Inflammation Modulation

Persistent inflammation is a major characteristic of chronic wounds and skin hypertrophic scars. MSCs have been shown to exert immunomodulatory effects on the inflammatory cells, mainly through paracrine signaling during wound healing. Previous studies have confirmed that MSCs can suppress T-cells, activate macrophages, and potentially recruit neutrophils, which are key mechanisms in the reduction of the inflammatory reaction [44]. MSCs also have antibacterial effects, which serve as another mechanism to reduce an excessive inflammatory reaction [45, 46].

During the transition from the inflammatory stage to the next stage of wound healing, macrophages undergo a change toward a type 2 inflammatory phenotype, which is characterized by increasing levels of anti-inflammatory cytokines and a simultaneous decrease in the levels of inflammatory cytokines [47–49]. In addition, the macrophages also influence wound healing in a positive way by decreasing the numbers of bacteria, increasing angiogenesis, providing matrix deposition, and producing the myriad of growth factors that are necessary to activate the keratinocytes, fibroblasts, and ECs [50]. However, in a persistently inflammatory environment, the macrophages are dysregulated and become skewed toward a type 1 inflammatory phenotype, impeding progress toward wound reepithelialization.

The inflammatory environment of the wound activates the MSCs to initiate their immunomodulatory effects. First, MSCs regulate macrophages through a paracrine-like reprogramming of inflammatory type 1 macrophages to a type 2 anti-inflammatory phenotype. Activated macrophages (type 2 macrophages) have been shown to decrease their expression of inflammatory cytokines and increase anti-inflammatory signaling [51]. Second, MSCs suppress T cell proliferation to reduce wound site inflammation: this process depends on MSC-mediated induction of IL-10 in T cells and macrophages. In addition, MSCs modulate TNF-*α* production to attenuate the excessive inflammatory effects and reduce NK cell function in the inflammatory phase, lowering IFN-*γ* activity in the process [52]. Finally, MSCs provide antiscarring properties by secreting prostaglandin E2 (PGE2), which induces the increased expression of IL-10 by T cells and macrophages. IL-10 exerts antiscarring effects by downregulating TGF-*β*1 expression, reprogramming wound fibroblasts to favor ECM remodeling, and decreasing the expression of inflammatory cytokines (such as IL-6 and IL-8) to prevent an excessive increase of collagen deposition in the wound [24, 53]. The antibacterial effects of MSCs have also been verified and are critical for reducing excess inflammation in the wound. MSCs exert antibacterial activity directly by secreting antibacterial factors such as LL-37 [46] and indirectly by enhancing the phagocytosis by immune cells [54].

Taken together, the effects produced by MSCs may help to solve the problem of unmitigated inflammation at the wound site and promote the completion of wound reepithelialization.

### 4.2. Repairing Cell Dysfunction

Wound reepithelialization mainly relies on the migration, proliferation, and differentiation of keratinocytes and their cross-talk with fibroblasts, ECs, and other skin cells. Dysfunction of any of these cells leads to an inhibition or delay of the reepithelialization phase. MSCs participate in reepithelialization through transdifferentiation into multiple skin cells including keratinocytes and ECs and by secreting various types of cytokines that promote cell survival, proliferation, and differentiation [55, 56].

Many studies have demonstrated that MSCs can differentiate into keratinocytes in a specific environment to promote reepithelialization [57–60]. Chen et al. showed that the GFP + MSCs used to treat murine wounds are capable of differentiating into keratinocytes that can regenerate the epidermis in vivo [58]. Sasaki et al. showed that when BMSCs were injected into the wounds of mice, the MSCs differentiated not only into keratinocytes but also into ECs and pericytes in vivo [61]. Moreover, the intravenous injection of allogenic BrdU-labeled BMSCs into full-thickness skin wounds in rats showed that the BrdU-labeled BMSCs could differentiate to ECs in the granulation tissue and to epidermal cells in the regenerated skin. These results showed a significant enhancement of the speed of reepithelialization, the number of epidermal ridges, and the thickness of the regenerated epidermis [59]. These findings suggest that MSCs can differentiate into multiple skin cells including keratinocytes that regenerate the skin epidermis, which contributes to the reepithelialization of the wound.

In addition to the differentiation of MSCs to keratinocytes, their secretory function also plays an essential role in the reepithelialization process. MSCs in a cutaneous wound release growth factors including IGF-1, EGF, KGF, and mitogens that promote the proliferation of fibroblasts, keratinocytes, and ECs in vitro. Studies have shown that adipose-derived stem cells (ASC) accelerated the reepithelialization of cutaneous wounds via the paracrine secretion of a various growth factors, including PDGF, basic fibroblast growth factor (bFGF), and TGF-*β* [62, 63]. In addition to accelerating reepithelialization, MSCs have been shown to improve the quality of the epidermis. MSCs enhance the proliferation of endogenous keratinocytes and increase the number of appendage-like structures [57]. These cells were shown to promote reepithelialization through paracrine signaling that accelerated wound healing.

Revascularization is a necessary part of the overall wound healing process that provides nutrients and oxygen to a significant proportion of the cells that regulate wound healing. Under normal circumstances, injury stimulates the proliferation of ECs, but some harmful stimuli including diabetes and burns often lead to inhibition of EC proliferation and increased apoptosis. MSCs can differentiate into ECs to yield new vessels and produce a number of proangiogenic factors that help to reestablish the blood supply to the wound bed. The most notable of these factors is VEGF, a potent stimulator of angiogenesis that is regulated by IL-6 and TGF-*α* [51, 61, 64]. In addition, studies have demonstrated that MSC-conditioned medium increased angiogenesis in vitro. Skin wounds treated with BM-MSC-conditioned medium had increased numbers of cells positive for CD34, Flk-1, or C-kit, markers of endothelial lineage cells, suggesting an increased recruitment of ECs and EPCs into the wound [57].

These recent studies have demonstrated that MSCs can repair the function of keratinocytes and ECs, the predominant cells in the reepithelialization process, by processes including increasing the proliferation of keratinocytes and ECs and decreasing apoptosis through paracrine signaling, as well as by directly differentiating into keratinocyte and ECs to promote reepithelialization and achieve wound healing.

### 4.3. Antiscarring

Ideally, the wound repair process proceeds in a regulated fashion in which a balance between collagen synthesis and degradation is maintained [65]. However, in many situations, skin wound repair becomes seriously dysregulated, resulting in the formation of hypertrophic scars that are characterized by excessive collagen deposition and excessive myofibroblast proliferation in the dermal tissue [53].

MSCs play a pivotal role in skin hypertrophic scarring. Mobilization and homing of MSCs to the injury site are involved in wound healing. These processes inhibit the formation of scars in skin wounds by inhibiting excessive inflammation and producing antifibrotic factors. Many recent studies have demonstrated that MSCs migrate to wound sites and participate in attenuating inflammation and reprogramming the resident immune and wound-healing cells in the wound to favor skin regeneration and inhibit scar formation. Wu et al. have shown that the administration of BMSCs could suppress bleomycin-induced skin fibrosis formation. This study showed that BMSCs can downregulate antifibrotic factors to alleviate inflammation and attenuate myofibroblast proliferation and differentiation as well as reducing collagen deposition and matrix production to favor the remodeling of the ECM [53]. Liu et al. have shown that treatment of a rabbit hypertrophic scar with human BMSCs efficiently regulated inflammation and prevented scar formation. In this study, the therapeutic effects of the hMSCs were attributed to the secretion of the anti-inflammatory protein, TNF-alpha-stimulated protein 6 (TSG-6) [66].

Collectively, these data demonstrate that MSC treatment can reduce skin hypertrophic scar formation to improve skin reepithelialization.

### 4.4. Crosstalk between the Cells and Microenvironment

The reepithelialization of a wound requires coordinated interactions among inflammatory cells, keratinocytes, fibroblasts, vascular endothelial cells, and immune cells as well as the local microenvironment. Keratinocyte migration and proliferation rely on the interactions of the keratinocytes with the fibroblasts and the ECM and on a great diversity of paracrine factors present in the wound site [67]. During reepithelialization, endogenous epithelial stem cells contribute to the reepithelialization by differentiating into epithelial cells. In addition, basal keratinocytes at the wound margins migrate under the fibrin clot to participate in reepithelialization [68].

Keratinocytes in the epidermis of hypertrophic scars become activated and produce growth factors that affect the inflammatory response, endothelial cells, and fibroblasts. The persistence of activated keratinocytes implies abnormal keratinocyte migration and proliferation and abnormal epidermal-mesenchymal interactions that delay the reepithelialization [25]. In chronic wounds, the decreased migration, proliferation, and increased apoptosis of keratinocytes and fibroblasts impede the reepithelialization.

The therapeutic effects of MSCs are due to their ability to modulate the surrounding environment and activate endogenous progenitor cells [69, 70]. After a skin injury, MSCs migrate into the wound site and contribute to angiogenesis and keratinocyte proliferation and migration by secreting paracrine factors [68]. Studies also showed that MSCs-conditioned medium (MSCs-CM) improves keratinocyte proliferation and migration in an inflammatory microenvironment [71, 72]. The MSCs-CM was sufficient to stimulate macrophage and endothelial migration and enhance reepithelialization in vivo [51]. Furthermore, the delayed reepithelialization in chronic wounds increases the chance of infection. Under these conditions, the bacteria can inhibit keratinocyte proliferation and migration and increase keratinocyte death [68]. MSCs have antibacterial effects and enhance skin reepithelialization.

### 4.5. Tissue-Engineered Skin Constructs

Tissue-engineered skin composed of skin keratinocytes and fibroblasts has been widely used in skin regeneration [63]. However, the therapeutic application of this technique is hampered by insufficient resources, limited proliferative capacity, immunological rejection, and terminal differentiation [44]. Therefore, these seed cells are not ideal for skin tissue engineering. Moreover, the lack of specific surface antigens on the epidermal stem cells makes them difficult to purify and culture or amplify. For these reasons, these cells are not widely applied in skin tissue engineering.

MSCs are widely used as an appropriate source of seed cells for tissue-engineered skin due to their pluripotency, self-renewal capacity, and low immunogenicity. Recent studies have focused on the application of MSCs in the construction of tissue-engineered skins. For example, MSCs applied topically as cell sheets to full-thickness murine wounds have demonstrated improved healing by differentiation toward endothelial and epidermal lineages [73]. In another study, human BMSCs were cultured on a gelatin scaffold with pNIPAAm [poly(N-isopropylacrylamide)] and transplanted as grafts for skin regeneration. The expression of human pan-keratin and cadherin, which are indicators of epithelial regeneration, was significantly increased after transplantation. This observation indicated that BMSCs can differentiate into epidermal cells and complete reepithelialization [74]. In our recent study, we successfully constructed epidermal substitutes through the culture of umbilical cord MSCs (UC-MSCs) on the surface of collagen-chitosan scaffolds at an air-liquid interface to induce epidermal differentiation. Animal experiments showed that the epidermal substitutes promoted wound healing by enhancing epidermal and hair follicle regeneration [44]. Interestingly, BMSCs can also be used as a feeder layer to promote autologous keratinocyte expansion for the preparation of epidermal sheets for burns [7]. These studies have shown that MSCs can be used as ideal seed cells for the construction of skin substitutes and that the MSCs can restore the properties of the epidermis and promote rapid skin reepithelialization.

### 4.6. Clinical Trials

These studies have shown that MSCs promote reepithelialization and accelerate cutaneous wound repair through various mechanisms. Several clinical trials in which MSCs were applied in studies of wound reepithelialization have demonstrated that MSCs promote skin reepithelialization through paracrine signaling and differentiation [75]. In 2008, Yoshikawa and colleagues treated 20 patients with various nonhealing wounds with autologous BM-MSC-composite grafts. The results showed that 18 of the 20 wounds completed reepithelialization, as confirmed by histological examination [76]. Similarly, the use of a collagen scaffold seeded with allogeneic nondiabetic MSCs to treat nonhealing diabetic foot ulcers resulted in increased angiogenesis and improved reepithelialization. Clinical trials in patients with chronic wounds revealed a reduction of ulcers and reepithelialization when MSCs were applied [77]. These clinical trials showed the potential benefit of MSCs in completing reepithelialization to achieve wound healing, including an improved average rate of reepithelialization and epidermal quality after treatment by mechanisms including paracrine signaling, epithelial differentiation, and angiogenesis.

## 5. Questions and Future Perspectives

These studies on MSC-facilitated skin reepithelialization through cell proliferation and differentiation the secretion of growth factors, and as a source of seed cells in skin tissue engineering have all shown promising results. However, many questions remain. Future directions for research in this field should focus on optimizing the function of MSCs in the reepithelialization process to maximize the regenerative properties of MSC-based therapies. The optimization should include designing appropriate delivery systems and extending cell survival through independent technologies or in combination with these delivery systems [78]. The clinical effectiveness of MSC-based therapies is dependent on the number of cells delivered and their survival, so it is important to optimize the delivery procedure. Current research indicates that the contribution of the MSCs is limited by poor engraftment and survival of the MSCs at the wound site. In addition, many studies strongly imply that microenvironmental cues have a critical influence on MSCs activity and fate. Therefore, the materials and resident cells that contribute to a microenvironment that favors the survival and differentiation of MSCs needs to be explored more extensively [79]. Furthermore, manipulation of the MSCs before their use in a therapeutic application may be appropriate. For example, methods to induce the MSCs to differentiate into special cells that are more conducive to improving reepithelialization [80] or addition of an adjunctive therapy (X factor) into the MSC-based therapy, that is, the treatment of BMSCs with some chemical compound, may be of value. Clearly, the X factor should be safe, effective, and economic for the application in a clinical setting [81].

Addressing these questions will determine whether MSC-based therapies can be used to promote the successful completion of reepithelialization that is impaired during abnormal wound healing, including chronic wounds and hypertrophic scars.

## 6. Conclusion

Reepithelialization is achieved by a coordinated interaction of keratinocyte migration, proliferation, and differentiation during wound repair. On the basis of their extensive capabilities and their effects on reepithelialization, MSCs seem to have a significant impact on the treatment of chronic nonhealing wounds or hypertrophic scars associated with impaired reepithelialization. We have reviewed the recent studies that analyzed the participation of MSCs in the impaired reepithelialization process along with their roles in wound healing, which include the inhibition of an excessive inflammatory response, secretion of important factors, differentiation into multiple skin cell types, and recruitment of other host cells. In addition, several preclinical and clinical studies investigating the potential of MSCs in wound healing have highlighted the role of these cells in enhanced reepithelialization. Although the function of MSCs in the reepithelialization process has shown promising results including well-organized epidermal regeneration and good quality reepithelialization, questions regarding the efficacy of MSCs in cell therapy remain to be resolved.

## 7. Comments

Our review shows that BMSCs participate in the reepithelialization through several mechanisms including differentiation and secretion of paracrine factors. A previous study showed that less than 1% of the BMSCs differentiate into keratinocytes in the absence of skin injury. After skin injury, engraftment of BMSCs as keratinocytes increased within 1 day and continued to increase to approximately 4% by 3 weeks. In acute wounds, the BMSCs transdifferentiation is probably not disturbed. In chronic wounds, BMSCs may differentiate into multiple skin cell types to promote reepithelialization, and in larger epidermal loss, the participation of BMSCs seems to be indispensable, implying a profound therapeutic potential of these cells for skin wounds. However, it appears that some factors in chronic wound sites prevent the BMSCs from migrating into the wound site and from further differentiation into keratinocytes. Therefore, a microenvironmental niche in which the cells reside appears to be required to promote differentiation into specific cell types, and unfortunately there are no detailed data to define this niche.

In addition, although increasing numbers of studies have confirmed the important role that MSCs play in the reepithelialization process, it is still unclear how effectively MSCs contribute to reepithelialization via specific transdifferentiation. This may be partly due to poor engraftment and the survival of MSCs at the wound site. Thus, extending cell survival might induce more cells to undergo specific differentiation, resulting in better functional organization of the skin wounds. Although the transdifferentiation mechanism of MSCs has been extensively investigated, understanding of this mechanism is still insufficient, and further study is needed before these cells can be used in clinical applications.
